# The relation between EQ-5D and fatigue in a Dutch general population sample: an explorative study

**DOI:** 10.1186/s12955-021-01771-3

**Published:** 2021-04-29

**Authors:** I. Spronk, S. Polinder, G. J. Bonsel, M. F. Janssen, J. A. Haagsma

**Affiliations:** 1 Department of Public Health, Erasmus MC, University Medical Center Rotterdam, P.O. Box 2040, 3000 CA Rotterdam, The Netherlands; 2 Association of Dutch Burn Centres, Maasstad Hospital, Rotterdam, The Netherlands; 3 EuroQol Group Executive Office, Rotterdam, The Netherlands; 4 Section Medical Psychology and Psychotherapy, Department of Psychiatry, Erasmus MC, Rotterdam, The Netherlands

## Abstract

**Background:**

Fatigue negatively influences health-related quality of life. It is questionable whether fatigue is sufficiently covered by the EQ-5D. This study investigated whether fatigue is covered by the existing domains of the EQ-5D.

**Methods:**

A Dutch general population sample completed the EQ-5D (3L and 5L version) and the Rivermead Post-Concussion Symptoms Questionnaire (RPQ), of which the fatigue item was used. Outcomes were compared between participants with and without a chronic health condition. Convergent validity was assessed, and multivariate regression analyses was used to predict the RPQ fatigue item from the EQ-5D-3L and EQ-5D-5L domains separately.

**Results:**

3027 people completed the survey, of whom 52% had ≥ 1 chronic health condition. Fatigue was reported by 48% of the participants. Fatigue was moderately correlated to the EQ-5D domains ‘pain/discomfort’, ‘usual activities’, and ‘anxiety/depression’ for the 3L (r = 0.379–0.426) and 5L version (r = 0.411–0.469). For the 5L, also a moderate correlation with ‘mobility’ (r = 0.335) was observed. The remaining correlations were weak. All EQ-5D-3L and 5L domains except for ‘mobility’ were significantly associated with the RPQ fatigue item (unstandardized Beta = − 0.20–0.67; *p* < 0.01 to *p* = 0.04). Comparable outcomes were found for participants with and without ≥ 1 chronic health condition.

**Conclusions:**

The extent to which fatigue is covered by the EQ-5D domains is small to moderate, with the EQ-5D-5L being slightly more sensitive to capture fatigue compared to the EQ-5D-3L. An extra fatigue item for the EQ-5D may add value, as fatigue is not fully captured by the existing domains, both in people with and without a chronic health condition.

## Introduction

The impact of a health condition, the effectiveness of treatments and interventions, and the level of quality of care are increasingly evaluated by assessment of health-related quality of life (HRQL) of patients [1–3]. HRQL reflects patients’ perceptions of their condition on physical, psychological and social wellbeing [4]. HRQL instruments are developed as generic (i.e. applicable to any health condition) or disease-specific measures. Generic instruments facilitate comparison between different health conditions, whereas disease-specific instruments take the effects of a specific health condition into account in more detail [5].

A widely used generic HRQL instrument is the EQ-5D [6]. Its descriptive system consists of five domains: mobility, self-care, usual activities, pain/discomfort and anxiety/depression, which can be scored on a three or a (more refined) five level ordinal scale (EQ-5D-3L or EQ-5D-5L) [6, 7]. The instrument is cognitively simple, has good feasibility and only takes a few minutes to fill out [8]. Due to its conciseness the instrument is not comprehensive in all condition areas or populations; it does not capture all (disease-specific) aspects of health [9, 10]. This may lead to a lack of content validity and scale sensitivity [9, 11–13].

It is questionable whether fatigue is sufficiently covered by the EQ-5D. Fatigue is a sequela of a lot of chronic health conditions and has a considerable impact on HRQL [14–17]. An energy/tiredness domain was considered during development of the EQ-5D, but not included in the final version of the instrument as it was concluded that it had no significant additional effect in small-sized analysis [18–20]. However, more recent studies suggest that adding a tiredness/fatigue domain to the EQ-5D is valuable [21–25]. For example, a study by Efthymiadou et al. among 767 patient representatives from 38 countries showed that 17 important aspects were not captured by the EQ-5D, with fatigue being the most mentioned aspect [25].

Before testing the addition of an extra fatigue domain, it should be studied whether, and if so, to what extent, fatigue is captured by the five existing EQ-5D domains. In order to do that, one or more populations in which fatigue is prevalent is needed. In the general population prevalence rates of fatigue up to 50% have been reported [26–28]. These relatively high fatigue rates show that the general population is a suitable sample to investigate whether fatigue is captured by the EQ-5D instrument or represents a distinct piece of health information. Studies on the measurement properties of the EQ-5D-3L and EQ-5D-5L have shown that the EQ-5D-5L has a higher sensitivity and precision in health state measurement compared to the EQ-5D-3L [29, 30], though the EQ-5D-3L version is still often used. Therefore, the primary aim of this study was to explore whether fatigue is covered by the existing domains of the EQ-5D. The EQ-5D-3L and the EQ-5D-5L are separately tested as sensitivity of the latter is higher. Because fatigue is often related with having a chronic health condition [24–27], the secondary aim was to study whether outcomes differ between subgroups of people with and without a chronic health condition.

## Methods

### Participants

During the period June 29th till July 31st 2017, Survey Sampling International recruited participants [31]. They distributed and launched a survey in an existing large Dutch internet panel. The selected sample was representative of the population aged 18–75 with respect to age, sex and educational level. Informed consent for this survey was obtained from all members that agreed to fill in the survey. This study was part of the Collaborative European NeuroTrauma Effectiveness Research (CENTER-TBI) study (EC Grant 602150), a European multicentre prospective cohort study on the impact of traumatic brain injury (TBI). The data of the general population sample (used in present study) were to be used as reference data for the assessment of post-injury impact. Ethical approval was obtained from the Leids Universitair Centrum—Commissie Medische Ethiek (approval P14.222/NV/nv). Only data from participants who completed the full survey were included in the analysis.

### Measures

The survey included questions on socio-demographic information. Participants provided information regarding their age, gender, area of residence, educational level, household income level. Level of education was measured as the highest level achieved and coded based on the International Standard Classification of Education (ISCED) into three groups: up to lower secondary education (‘low’), completed upper secondary education (‘middle') and tertiary education (‘high’) [32]. Medical information included the presence of self-reported chronic health condition(s), including: asthma, chronic bronchitis, severe heart disease, consequences of a stroke, diabetes, severe back complaints, arthrosis, rheumatism, cancer, memory problems due to a neurological disease/dementia, memory problems due to ageing, depression or anxiety disorder, and/or other chronic health conditions.

The survey also included both the EQ-5D-3L and the EQ-5D-5L for all respondents [6, 7]. It was randomly assigned which version was administered first. The EQ-5D asks about your health today; the domains included are: mobility, self-care, usual activities, pain/discomfort, and anxiety/depression. The EQ-5D-3L offers three response options (no problems, moderate problems, and extreme problems/unable to) [6]. The EQ-5D-5L offers five response options (no problems, slight problems, moderate problems, severe problems, and extreme problems/unable to) [7]. Based on the five domains, an EQ-5D value or utility score (through weighting) was calculated based on Dutch value sets, separately for the EQ-5D-3L and EQ-5D-5L [33, 34]. The utility score ranges between 0 (referring to a state as bad as being dead) and 1 (referring to full health), with negative values for health states considered worse than death [8].

The survey also included the Rivermead Post-Concussion Symptoms Questionnaire (RPQ) [35]. This questionnaire assesses sixteen different symptoms. One of these items is about fatigue: ‘Do you (i.e., over the last 24 h) suffer from fatigue?’. Answer options included 0 (not experienced at all), 1 (no more of a problem), 2 (a mild problem), 3 (a moderate problem) and 4 (a severe problem).

### Data analyses

We used IBM SPSS Statistics 25 for all analyses. Descriptive statistics were used to assess the participant characteristics and outcomes of the EQ-5D-3L domains, EQ-5D-5L domains, and the RPQ fatigue item. Data are shown for the overall sample, as well as for subgroups based on whether or not participants had a chronic health condition. EQ-5D and fatigue level scores were compared across groups with Mann Whitney U tests for ordinal variables and with chi-square tests for categorical variables.

We head-to-head compared the EQ-5D-3L and EQ-5D-5L domains with the RPQ fatigue item. The proportion of participants with corresponding answers was assessed and tested using a chi-squared test. Corresponding answers were defined as reporting problems on both the EQ-5D domains and the RPQ fatigue item, or reporting no problems on any instrument. For example, a person reported a corresponding answer for the domains EQ-5D mobility and RPQ fatigue item if he/she reported mobility and fatigue problems, i.e. an EQ-5D mobility score > 1 and a RPQ fatigue item score > 1.

The Spearman rank correlation coefficient was used to study rank order correlations between the EQ-5D domains and the RPQ fatigue item, both in the total sample, and in the subgroups of people with and without a chronic health condition [36]. Cohen’s criteria were applied to evaluate the strength of association: correlations were strong if r ≥ 0.50, moderate if r ≥ 0.30–0.49, and weak if r ≥ 0.10–0.29 [37]. Next, it was measured to what extent variability in fatigue was captured by the EQ-5D domains. We applied multivariate regression analyses to explore which EQ-5D-3L and EQ-5D-5L domains associated with the RPQ fatigue, with relevant participant characteristics added (sex, age, level of education, work status, income and the presence of a chronic health condition). The significance level for all explorative analyses was set at *p* < 0.05.

## Results

### Participants

A total of 3564 persons returned the questionnaire, of whom 3027 (85%) fully completed it. Participants were on average 44.7 years old (SD 15.3) and 50% was male (Table 1). About half of the participants had a middle level of education (47%), and was employed (54%). Half of the participants had one or more chronic health condition (52%).

**Table 1:** Characteristics of study population

Characteristic	Total sample (n = 3027)
Sex: Male	1520 (50.2%)
Age (M, SD)	44.7 (15.3)
Age categories	
18 to < 25 years	365 (12.1%)
25 to < 40 years	814 (26.9%)
40 to < 60 years	1231 (40.7%)
60–75 years	617 (20.4%)
Level of education	
Low	811 (26.8%)
Middle	1420 (46.9%)
High	796 (26.3%)
Work status^a^	
Employed	1635 (54.0%)
Unemployed	316 (10.4%)
Looking after others	125 (4.1%)
Student	209 (6.9%)
Retired	386 (12.8%)
Unable to work	356 (11.8%)
Household income^b^	
Low	540 (17.8%)
Middle	1270 (42.0%)
High	555 (18.3%)
Do not know/do not want to tell	662 (21.9%)
Number of chronic health conditions	
No disease	1453 (48.0%)
1 disease	971 (32.1%)
2 diseases	368 (12.2%)
3 diseases	149 (4.9%)
≥ 4 diseases	86 (2.8%)

^a^Work status was categorized as employed (employee and self-employed), unemployed (consisting out of work for more than and less than 1 year), looking after others (e.g. a carer or parent), a student, retired and unable to work

^b^Income was grouped as low (less than €20.000), middle (€20.000–€49.999) and high (more than €49.999)

### EQ-5D-3L, EQ-5D-5L, and fatigue outcomes

**Fig. 1:**
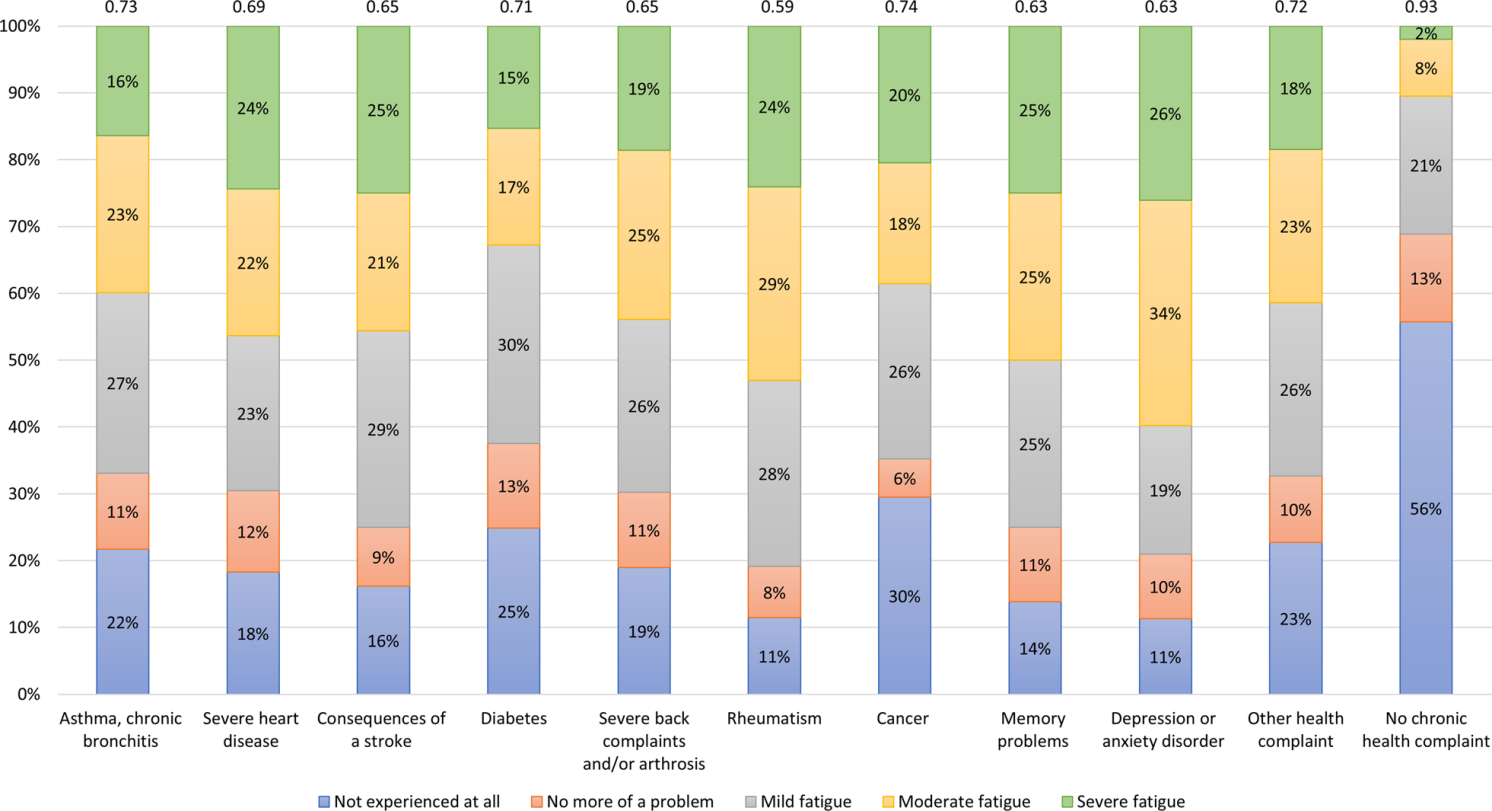
Frequency of responses on the RPQ fatigue item, according to the presence of a specific chronic health condition, including mean EQ-5D-5L utility score outlined above the bars

The mean EQ-5D utility score was 0.82 (SD 0.23) based on the EQ-5D-3L, and 0.83 (SD 0.21) based on the EQ-5D-5L (Table 2). Most problems were reported on the pain/discomfort domain: respectively 46% and 51% of the participants reported problems on the domain based on the EQ-5D-3L and EQ-5D-5L. A total of 23% of the participants reported mild fatigue, 16% moderate fatigue, and 9% severe fatigue. Participants with ≥ 1 chronic health condition had significantly worse outcomes on all EQ-5D domains and the RPQ fatigue item (Table 2). A total of 236 participants (15%) with ≥ 1 chronic health condition reported severe fatigue, whereas only 29 participants (2%) without a chronic health condition reported severe fatigue. Participants with rheumatism experienced fatigue most often (81%), followed by participants with depression or anxiety disorder (79%) (Fig. 1). Moderate to severe fatigue (RPQ fatigue item ≥ 3) was most often reported by participants with depression or anxiety disorder (60%), followed by participants with rheumatism (53%), and participants with memory problems (50%). Mean EQ-5D-5L utility score ranged between 0.59 for participants with rheumatism to 0.93 for participants without any chronic health condition (Fig. 1).

**Table 2:** EQ-5D-3L, EQ-5D-5L and RPQ fatigue outcomes for the total sample and separately for those with and without a chronic health condition

	Total sample (n = 3027)	Without a chronic health condition (n = 1453)	With ≥ 1 chronic health condition (n = 1574)
EQ-5D-3L			
Mobility (% with problems)	22.6%	6.3%*	37.5%*
Self-care (% with problems)	7.2%	1.7%*	12.3%*
Usual activities (% with problems)	25.3%	5.8%*	43.3%*
Pain/discomfort (% with problems)	46.0%	21.1%*	68.9%*
Anxiety/depression (% with problems)	27.2%	14.9%*	28.6%*
Utility score (M, SD)	0.82 (0.23)	0.93* (0.13)	0.72* (0.25)
EQ-5D-5L			
Mobility (% with problems)	27.1%	8.5%*	44.2%*
Self-care (% with problems)	8.7%	1.9%*	15.0%*
Usual activities (% with problems)	30.3%	8.0%*	50.9%*
Pain/discomfort (% with problems)	51.4%	27.8%*	73.3%*
Anxiety/depression (% with problems)	33.0%	18.4%*	46.4%*
Utility score (M, SD)	0.83 (0.21)	0.93* (0.11)	0.73* (0.23)
RPQ fatigue			
Not experienced at all	39.5%	55.7%*	24.5%*
No more of a problem	12.6	13.1%*	12.1%*
Mild fatigue	23.4%	20.6%*	25.9%*
Moderate fatigue	15.8%	8.5%*	22.6%*
Severe fatigue	8.8%	2.0%*	15.0%*

*Statistically significantly different between subgroups with and without a chronic health condition (*p* < 0.001)

### Head-to-head comparison EQ-5D-3L, EQ-5D-5L, and RPQ

#### EQ-5D-3L domains and the RPQ fatigue item

**Fig. 2:**
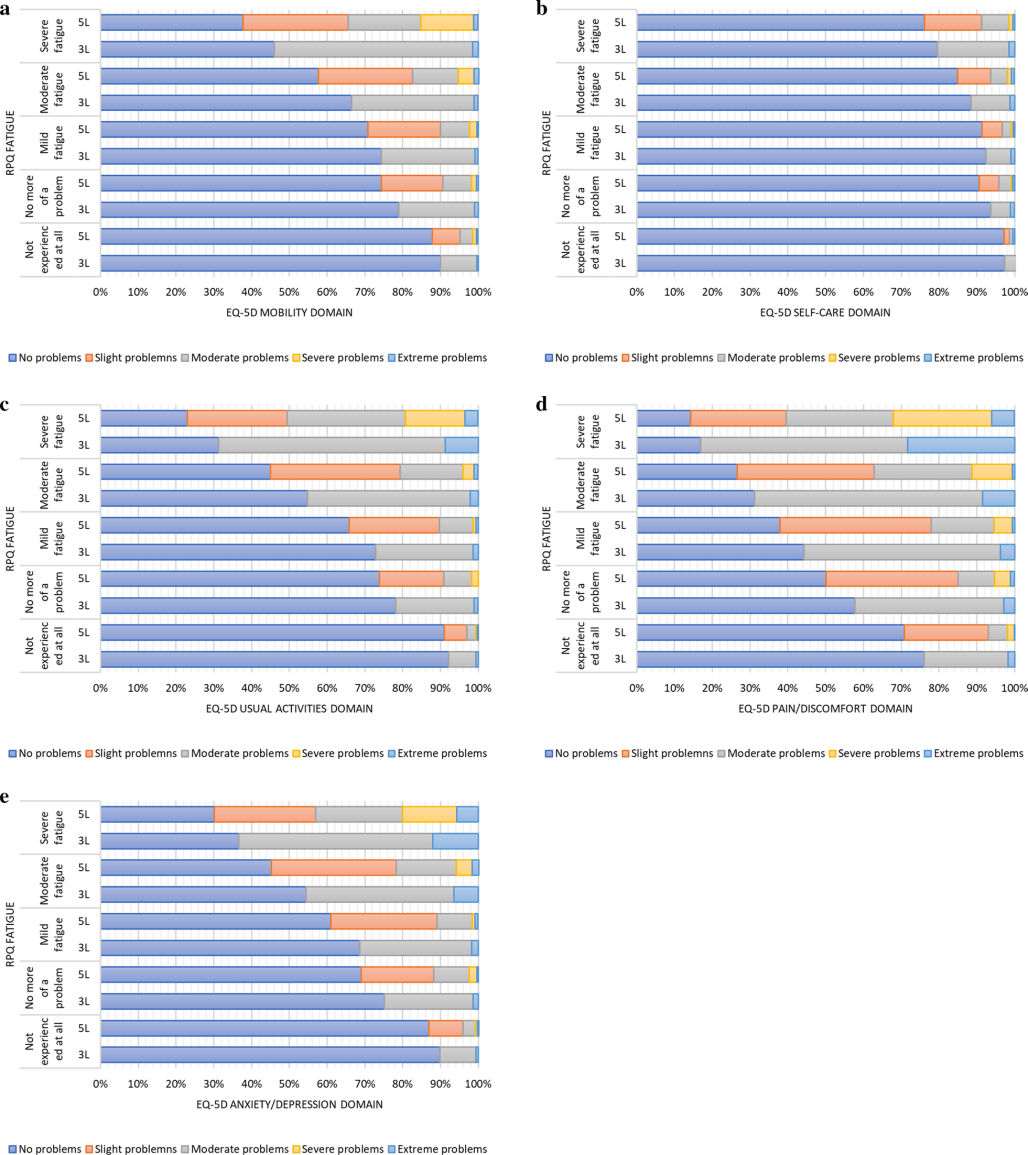
**a**–**e** Frequency of responses on the five EQ-5D-3L and the EQ-5D-5L domains, separately for all answer options of the RPQ item

For all domains, more than half of the participants (56–69%) reported corresponding answers (e.g. reporting problems on an EQ-5D domain and on the RPQ fatigue item) (Table 3). Most corresponding answers were reported on the ‘pain/discomfort’ domain and the RPQ fatigue item (69%). In case of non-corresponding answers/responses, most participants reported fatigue problems and no problems on the EQ-5D-3L domains. Chi-square test showed that answers on all EQ-5D domains were related to the RPQ fatigue item (all *p* < 0.001). The distribution of answer options of each EQ-5D domain for each level of fatigue is presented in Fig. 2. It graphically presents an increasing percentage of participants reporting problems on the EQ-5D domains with higher fatigue levels. This is especially seen for the ‘pain/discomfort’ domain, and the ‘usual activities’ domain. The figure also depicts the differences between the EQ-5D-3L and EQ-5D-5L, with a higher percentage of participants reporting problems on the EQ-5D-5L compared to the EQ-5D-3L for all levels of fatigue and on all domains of the EQ-5D.

**Table 3:** Head-to-head comparison of outcomes of the EQ-5D-3L domains and the RPQ fatigue item, and EQ-5D-5L domains and the RPQ fatigue item

	Comparison with EQ-5D-3L		Comparison with EQ-5D-5L	
	RPQ fatigue item		RPQ fatigue item	
	Fatigue	No fatigue	Fatigue	No fatigue
EQ-5D domains				
Mobility problems				
No (EQ-5D L1)	31.9%	**45.5%**	28.9%	**44.0%**
Yes	**16.0%**	6.6%	**19.0%**	8.1%
Self-care problems				
No (EQ-5D L1)	42.6%	**50.2%**	41.5%	**49.8%**
Yes	**5.4%**	1.8%	**6.5%**	2.2%
Usual activities problems				
No (EQ-5D L1)	28.4%	**46.3%**	24.5%	**45.2%**
Yes	**19.5%**	5.8%	**23.4%**	6.9%
Pain/discomfort problems				
No (EQ-5D L1)	16.7%	**37.3%**	14.3%	**34.3%**
Yes	**31.2%**	14.8%	**33.6%**	17.8%
Anxiety/depression problems				
No (EQ-5D L1)	27.8%	**44.9%**	24.1%	**43.0%**
Yes	**20.1%**	7.1%	**23.9%**	9.1%

Values printed in bold are considered corresponding answers, whereas the values not printed in bold are considered non-corresponding answers

#### EQ-5D-5L domains and the RPQ fatigue item

Except for the ‘pain/discomfort’ domain, all domains had more corresponding answers based on the EQ-5D-5L than based on the EQ-5D-3L (Table 3). Most corresponding answers were reported on the ‘usual activities’ domain and the RPQ fatigue item (69%). In case of non-corresponding answers, most participants reported fatigue problems and no problems on the EQ-5D-5L domains, though these percentages were all lower than based on the EQ-5D-3L, meaning that more problems were reported on the 5L version of the instrument. Responses on all EQ-5D-5L domains were significantly related to the RPQ fatigue item (all *p* < 0.01). Figure 2 graphically demonstrates that more problems on the EQ-5D-5L domains correspond with more fatigue, with most problems reported on the ‘pain/discomfort’ domain.

### Correlation between the EQ-5D domains and the RPQ fatigue item

#### EQ-5D-3L domains and the RPQ fatigue item

Spearman rank correlation coefficients of the EQ-5D-3L domains and the RPQ fatigue item are shown in Table 4. Correlations were moderate between the RPQ item and the domains ‘pain/discomfort’ (r = 0.426), ‘usual activities’ (r = 0.412), and ‘anxiety/depression’ (r = 0.379), and weak between the RPQ item and the other domains. In the subgroup of participants with ≥ 1 chronic health condition, all correlations were substantially stronger compared to the subgroup without a chronic health condition.

**Table 4:** Spearman’s rank correlation of the EQ-5D-3L domains with the RPQ fatigue item, and the EQ-5D-5L domains with and the RPQ fatigue item

	Mobility	Self-care	Usual activities	Pain/ discomfort	Anxiety/ depression
RPQ fatigue item					
EQ-5D-3L					
All participants	0.297*	0.184*	0.412*	0.426*	0.379*
Subgroup without chronic health condition	0.086*	0.050	0.200*	0.266*	0.303*
Subgroup with ≥ 1 chronic health condition	0.230*	0.155*	0.359*	0.332*	0.326*
EQ-5D-5L					
All participants	0.335*	0.210*	0.469*	0.447*	0.411*
Subgroup without chronic health condition	0.123*	0.073*	0.246*	0.277*	0.333*
Subgroup with ≥ 1 chronic health condition	0.270*	0.171*	0.428*	0.360*	0.343*

*p < 0.05 for the correlation, based on Spearman’s correlation coefficient

#### EQ-5D-5L domains and the RPQ fatigue item

Also, all domains of the EQ-5D-5L were correlated with the RPQ fatigue item, with the correlations being stronger compared to the EQ-5D-3L domains. All domains were moderately correlated to the RPQ fatigue item (r = 0.335 – r = 0.469), except for the ‘self-care’ domain, which was weakly correlated (Table 4). And, as with the EQ-5D-3L, all correlations were weaker in the subgroup of participants without a chronic health condition compared to the subgroup of participants with a chronic health condition.

### Variability in fatigue and the EQ-5D-3L and EQ-5D-5L domains

#### EQ-5D-3L domains and the RPQ fatigue item

Multivariate regression analysis showed that the EQ-5D-3L domains ‘anxiety/depression’ (unstandardized Beta = 0.67; *p* < 0.001), ‘pain/discomfort’ (unstandardized Beta = 0.59; *p* < 0.001), ‘usual activities’ (unstandardized Beta = 0.56; *p* < 0.001), and ‘self-care’ (unstandardized Beta = − 0.18; *p* = 0.021) were significantly associated with the RPQ fatigue item. These domains explained 29% of the variance of fatigue. Addition of participant characteristics explained another 5% (R^2^ = 0.340) (“Appendix 1”). Females, older age, being unable to work, having a low or unknown income, and having ≥ 1 chronic health complaint were associated with increased fatigue, whereas being a student and being retired were associated with lower fatigue levels.

In respondents having ≥ 1 chronic health condition, the EQ-5D-3L domains ‘anxiety/depression’ (unstandardized Beta = 0.60), ‘usual activities’ (unstandardized Beta = 0.49), and ‘pain/discomfort’ (unstandardized Beta = 0.46) (all *p* < 0.01), whereas in respondents without a chronic health condition all EQ-5D-3L domains were significantly associated domains (unstandardized Beta = − 0.55 to 0.75; *p* < 0.01 to *p* = 0.04) with the RPQ fatigue item.

#### EQ-5D-5L domains and RPQ fatigue item

For the EQ-5D-5L, multivariate regression analysis showed that the domains, ‘usual activities’ (unstandardized Beta = 0.42), ‘anxiety/depression’ (unstandardized Beta = 0.39), ‘pain/discomfort’ (unstandardized Beta = 0.34), and ‘self-care’ (unstandardized Beta = − 0.21) were significantly associated with the RPQ fatigue item (all *p* < 0.01) and explained 31% of the variance of fatigue. With participants characteristics added in the model, 35% of the variance was explained (“Appendix 1”). Females, older age, being unable to work, having a low income, and having ≥ 1 chronic health complaint were associated with increased fatigue, whereas being a student and being retired were associated with less fatigue levels. In both the subgroup of participants with (unstandardized Beta = − 0.15 to 0.39) and without ≥ 1 chronic health condition (unstandardized Beta = − 0.36 to 0.51) the associations between the EQ-5D-5L domains and the RPQ fatigue item were comparable.

## Discussion

This study showed that a 5-level item on fatigue is only partially covered by the existing domains of the EQ-5D-3L and the EQ-5D-5L, most by the domains ‘pain/discomfort’ and ‘usual activities’ and least by the domain ‘self-care’. The EQ-5D domains and the fatigue item were moderately to weakly associated, with somewhat stronger associations when the EQ-5D-5L was used instead of the EQ-5D-3L. The associations between the EQ-5D domains and fatigue were higher in participants with ≥ 1 chronic health condition compared to participants without a chronic health condition.

The results from our study suggest that the extent to which fatigue is covered by the EQ-5D domains is small to moderate, indicating that adding an extra fatigue item to the EQ-5D might be considered. This is in line with earlier studies that suggested adding a fatigue (or related construct) item to the EQ-5D. Comparable constructs that were all related to being fatigued, but named differently were: energy/sleep [38], energy [10], fitness [24], fatigue [25], energy/fatigue [39], tiredness [21, 23]. These studies used different approaches to assess the potential need of a fatigue item for the EQ-5D, but all showed that adding a fatigue item has significant impact [10, 21, 23–25, 38, 39]. All these separate studies, including ours, provided indications on the potential value of adding a fatigue item to the EQ-5D, a so-called candidate bolt-on item. A ‘bolt-on’ is a specific domain that covers a specific health problem or dysfunction that has not sufficiently covered by the original instrument [40]. During the development of the EQ-5D, an energy/tiredness domain was considered, but eventually not included in the final version of the instrument as the added information—tested in small pilots, was small [18–20]. However, the time might be right to re-evaluate and further investigate this decision. As fatigue is an important sequela of many chronic conditions, studying the potential gain of adding a fatigue item to the EQ-5D is highly relevant. Additional analyses on the value of a fatigue bolt-on are valuable to conclude whether an extra domain captures additional information and improves the coverage of HRQL. We recommend that future studies investigate fatigue, with a suitable EQ-5D-5L response set, as a bolt-on item for the EQ-5D.

It is notable that our results showed that fatigue does not always result in problems on the existing domains of the EQ-5D, whereas problems on most EQ-5D domains did seem to result in fatigue. The other EQ-5D domains thus seem to be dominant over fatigue. Also, of note, is that a considerable part of the participants did report fatigue and no pain/discomfort problems, indicating that these participants did not consider fatigue as discomfort. Results of our study on the positive association between having a chronic health condition and experiencing fatigue are in line with earlier studies [24–27]. Study participants with one or more chronic health conditions reported significantly more frequently problems with fatigue. Fatigue is a sequel of many chronic health conditions, and is associated with lower HRQL [24–27]. Besides, our study showed that the EQ-5D-5L is somewhat more sensitive to capture fatigue compared to the EQ-5D-3L instrument, which is in congruence with current evidence. Earlier studies confirmed that the five level instrument is more sensitive to assess problem on the different domains and ceiling effects are substantially lower compared to the three level instrument [29, 41]. Despite the fact that the EQ-5D-5L instrument was somewhat more sensitive, results of all analyses were very consistent among the two versions of the instrument.The present study included some strengths and limitations. A strength included the large sample size that was representative of the Dutch population with respect to age, sex and educational level. Also, the prevalence of a chronic health condition was comparable to the Dutch population (52% vs. 58%) [42]. Other strengths include the ability to divide the sample in a group with and without a chronic health condition, and the high prevalence and wide range of fatigue scores which allowed us to study whether fatigue was captured by the EQ-5D instruments. By the inclusion of both the EQ-5D-3L and EQ-5D-5L instrument, we were able to compare outcomes between the EQ-5D-3L and EQ-5D-5L. However, this also could have been a limitation, as people became tired of completing comparable questions and just picked a box. We tried to avoid a systematic effect on the means by randomly assigning the EQ-5D-3L and EQ-5D-5L version first. Another limitation included the use of the RPQ fatigue item to assess fatigue. The RPQ instrument was originally developed for assessing post-concussion symptoms; it is not specifically developed to assess fatigue in the general population, though an earlier study showed its ability to assess fatigue in a general population [31]. Another apparent limitation was the difference in timeframe (your health today vs. fatigue in the past 24 h). Another limitation is the web-based administration of the survey without a predefined sampling frame. By this method, we were unaware of selective non-response and unable to study whether relations between 3L, 5L and fatigue were affected by particular respondents being overrepresented.

## Conclusion

This explorative study showed that the extent to which fatigue is captured by the EQ-5D domains is small to moderate in a sample of the general population, with the EQ-5D-5L being slightly to moderately more sensitive to capture fatigue compared to the EQ-5D-3L. An extra fatigue item for the EQ-5D may add value, potentially in the form of a ‘bolt-on’ item, as fatigue is not fully captured by the existing EQ-5D domains, both in people with and without a chronic health condition.

## Data Availability

Data available on request due to privacy/ethical restrictions.

## Appendix

See Table

**Table 5:** Multivariate model for the RPQ fatigue item, separately for the EQ-5D-3L and EQ-5D-5L

	EQ-5D-3L		EQ-5D-5L	
	Unstandardized B	*p* value	Unstandardized B	*p* value
Constant	− 0.405	0.004	0.243	0.003
EQ-5D domains				
Mobility	0.042	0.506	0.024	0.515
Self-care	− 0.098	0.210	− 0.149	0.002
Usual activities	0.430	< 0.001	0.317	< 0.001
Pain/discomfort	0.442	< 0.001	0.259	< 0.001
Anxiety/depression	0.558	< 0.001	0.336	< 0.001
Characteristics				
Sex (male)	− 0.329	< 0.001	− 0.346	< 0.001
Age	− 0.005	0.005	− 0.006	0.002
Level of education				
Low	0.089	0.142	0.080	0.181
Middle	0.081	0.112	0.066	0.188
High (ref)				
Work status				
Employed (ref)				
Unemployed	− 0.067	0.354	− 0.110	0.120
Looking after others	− 0.106	0.317	− 0.057	0.586
Student	− 0.202	0.027	− 0.268	0.003
Retired	− 0.258	0.001	− 0.241	0.002
Unable to work	0.234	0.002	0.152	0.048
Household income				
Low	0.169	0.022	0.194	0.007
Middle	0.099	0.096	0.088	0.128
High (ref)				
Unknown	0.143	0.033	0.116	0.079
Having ≥ 1 chronic health complaint	0.380	< 0.001	0.277	< 0.001
F value R-square	85.920 0.340	< 0.001	91.060 0.353	< 0.001

^5^.
